# Omnidirectional Continuous Movement Method of Dual-Arm Robot in a Space Station

**DOI:** 10.3390/s23115025

**Published:** 2023-05-24

**Authors:** Ziqiang Zhang, Zhi Wang, Zhenyong Zhou, Haozhe Li, Qiang Zhang, Yuanzi Zhou, Xiaohui Li, Weihui Liu

**Affiliations:** 1 Faculty of Materials and Manufacturing, Beijing University of Technology, Beijing 100124, China; 2 Beijing Key Laboratory of Long-Life Technology of Precise Rotation and Transmission Mechanisms, Beijing Institute of Control Engineering, Beijing 100094, China

**Keywords:** dual-arm robot, inside the space station, omnidirectional movement, motion planning, simulation analysis

## Abstract

The burgeoning complexity of space missions has amplified the research focus on robots that are capable of assisting astronauts in accomplishing tasks within space stations. Nevertheless, these robots grapple with substantial mobility challenges in a weightless environment. This study proposed an omnidirectional continuous movement method for a dual-arm robot, inspired by the movement patterns of astronauts within space stations. On the basis of determining the configuration of the dual-arm robot, the kinematics and dynamics model of the robot during contact and flight phases were established. Thereafter, several constraints are determined, including obstacle constraints, prohibited contact area constraints, and performance constraints. An optimization algorithm based on the artificial bee colony algorithm was proposed to optimize the trunk motion law, contact point positions between the manipulators and the inner wall, as well as the driving torques. Through the real-time control of the two manipulators, the robot is capable of achieving omnidirectional continuous movement across various inner walls with complex structures while maintaining optimal comprehensive performance. Simulation results demonstrate the correctness of this method. The method proposed in this paper provides a theoretical basis for the application of mobile robots within space stations.

## 1. Introduction

As human space exploration continues to advance, space robots capable of performing various operational tasks within a space station have emerged as a significant field of research [1,2]. Mobile space robots, compared to robots with fixed bases, can significantly enhance their operational capabilities. Numerous researchers have focused their studies on the movement methods of space robots, providing valuable insights for their application within a space station [3].

Numerous researchers have investigated ground mobile robots, focusing on common mobility modes such as wheel-type [4,5,6,7], track-type [8,9], and leg-type [10,11,12,13]. These mobility modes have also been applied to planetary exploration robots to accomplish various space tasks. For example, the German Aerospace Center and European Space Agency developed a wheeled humanoid space robot, providing preliminary insights for future manned Mars missions [14]. Chen et al. [15] proposed a chameleon-inspired multi-toe quadruped robot for planetary surface exploration. Each bionic foot possessed four toes to stabilize them on granular materials. Moreover, other planetary exploration robots employing rolling [16] and jumping [17] modes have also been studied. In a weightless environment, the focus of research is on free-flying robots. For instance, Zhang et al. [18] proposed a full-size and free-flying humanoid robot named Taikobot that aimed to assist astronauts in a space station. Taikobot adopted a compact and lightweight design to work in microgravity, which reduced launch costs and improved safety during human–robot collaboration. Chen et al. [19] developed a free-flying space with gecko-inspired adhesives to grasp and manipulate large items or anchor themselves on smooth surfaces, and the tests were conducted on the International Space Station (ISS). For on-orbit tasks outside the space station, a crawling mode is typically employed to prevent robots from detaching from the space station. Representative examples of these types of robots include the US Space Station Remote Manipulator System (SSRMS) [20], the Chinese Core Module Manipulator (CMM) [21], and the European Robotic Arm (ERA) [22].

Numerous space robots are equipped with two manipulators to complete a variety of operational tasks [23,24]. For instance, Yang et al. [3] designed a continuum-manipulator space robot (CMSR). The continuum manipulators can be used for various tasks, such as assembly and inspection. A robot can also utilize its two manipulators to achieve free-flying movement by interacting with the space station’s inner wall, thus mimicking the way astronauts maneuver within a space station. Jiang et al. [1] proposed a wide-ranging stable motion control method for robot astronauts in space stations based on human dynamics. The experimental results showed that the method is a highly competitive solution for robot astronauts with human-like moving capabilities in space stations. For this type of robot, motion planning for the robot’s two manipulators is essential [25]. A common method for implementing motion planning is to divide two manipulators into a leader manipulator and a follower manipulator. The motion planning for the leader manipulator is completed first, and the motion trajectory of the follower manipulator is deduced through constraints [26,27,28]. By integrating perceptual information, such as vision [29,30] and haptic feedback [31], and intelligent algorithms, such as reinforcement learning [32], the motion planning of the manipulators can be achieved, especially in replicating the movement characteristics of human arms [33,34]. Due to the complexity of the space station’s internal environment, obstacle avoidance in motion planning has emerged as a focal point of research in this field [35]. Dual-arm space robots must not only avoid collisions with the complex environment but also achieve self-obstacle avoidance [36,37,38]. Moreover, numerous researchers concentrate on the dynamic characteristics of dual-arm space robots, enabling them to simultaneously meet kinematic and dynamic constraints [39,40,41]. Through the aforementioned motion planning methods, robots can complete complex space tasks, such as assembly [23] and target acquisition [42,43].

Researchers continue to explore the use of robots within space stations. However, achieving continuous movement in a weightless environment remains a formidable challenge. Moreover, the complex structure of the space station’s inner walls and the high-performance demands placed on the robots further complicate the analysis. This paper introduces an omnidirectional continuous movement method and has the following key contributions.

(1) The paper proposes a movement method for a dual-arm robot that simulates the way astronauts use their arms to interact with the inner walls for locomotion. This approach allows the robot to transition between different inner walls. The two manipulators function as both operational and mobile systems, reducing the structural complexity;
(2) Based on the kinematic and dynamic models of the dual-arm robot in both contact and flight phases, this study proposes not only the contact points between manipulators and the inner wall, but also the trunk movement law and the driving torques, as optimization variables. It aids in enhancing the robot’s overall performance;
(3) This research presents multiple constraints, including obstacle constraints, prohibited contact area constraints, and performance constraints. To improve optimization efficiency under these constraints, an optimization algorithm based on the artificial bee colony algorithm (OA-ABC) is introduced.

The findings of this study provide a theoretical foundation for the practical application of robots within space stations.

The remainder of the paper is structured as follows. Section 2 introduces the omnidirectional continuous motion method for the robot. Section 3 presents an example to validate the effectiveness of the proposed method. Section 4 discusses the results derived from the study.

## 2. Methods

### 2.1. Research Objectives

**Figure 1:**
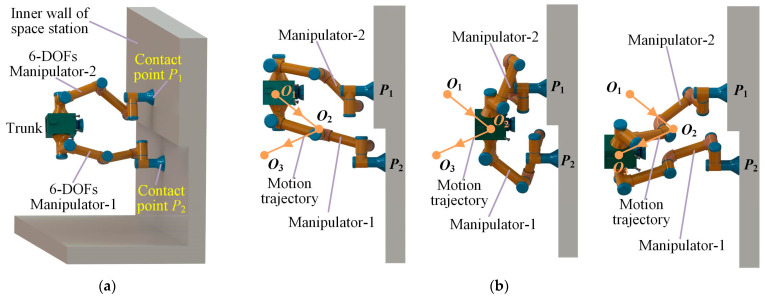
Diagram of dual-arm robot changing movement direction. (**a**) Diagram of the two manipulators in contact with the inner wall of the space station. (**b**) Omnidirectional movement process of space robot by using two manipulators.

In a space station, traditional movement modes, such as wheel-type and leg-type, are not applicable due to the low friction and complex surface structures of the inner wall. Astronauts often achieve efficient movement by using the reaction force between their arms or legs and the inner wall, thereby offering inspiration for the efficient movement of robots in a space station. As a solution, this paper proposes a dual-arm space robot, with each manipulator having six degrees of freedom (DOFs) and the capability to complete collaborative tasks. The robot can move through the interaction between the manipulators and the inner wall, as seen in Figure 1. Furthermore, the anthropomorphic mobile mode provides excellent environmental adaptability, enabling high mobility efficiency in an environment with obstacles.

The research objectives of this paper are as follows.

(1) The robot should achieve omnidirectional continuous movement in a space station using the manipulators. This means it should not have to stop and adjust its posture after each motion cycle (a motion cycle is defined as the process from the moment of contact between the robot and the inner wall to the next moment of contact with the inner wall);
(2) The robot should be capable of moving along an expected trajectory in a complex environment with obstacles, steps, and prohibited contact areas;
(3) The robot should possess superior comprehensive performance, including excellent dynamic stability, low energy consumption, and smooth motion laws.

### 2.2. Establishment of Mathematical Model

**Figure 2:**
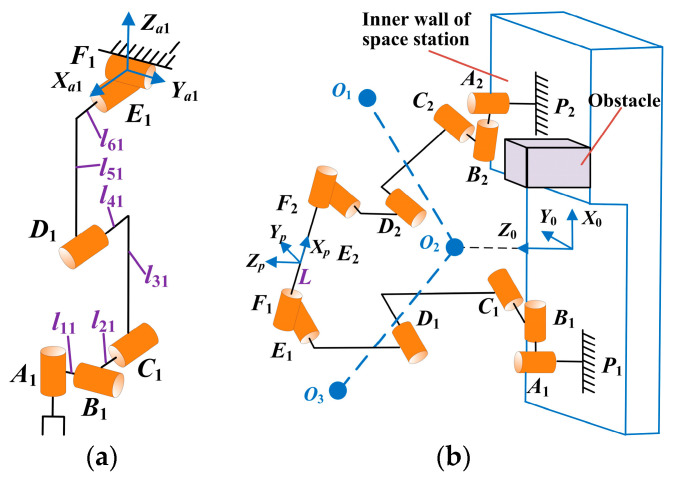
Mechanism diagram of dual-arm robot. (**a**) Mechanism diagram of 6 DOF manipulators. (**b**) Mechanism diagram of dual-arm robot in contact with the inner wall of the space station.

Figure 2 shows the mechanism diagram of the six DOF manipulators and the dual-arm robot in contact with the inner wall of the space station. In Figure 2b, *F*_1_*F*_2_ represents the trunk of the robot. *O*_1_*O*_2_ and *O*_2_*O*_3_ depict the potential trajectories of the trunk during the contact phase (the phase in which the two manipulators simultaneously make contact with the space station’s inner wall). These trajectories may be straight lines or curves. *O*_2_ is the ideal nearest point, but it may change according to actual circumstances. The coordinate origin of the fixed coordinate system *O*_0_–*X*_0_*Y*_0_*Z*_0_ coincides with the projection point of *O*_2_. The directions of the coordinate axes are demonstrated in Figure 2b. The coordinate origin of the moving coordinate system *O_p_*–*X_p_Y_p_Z_p_* coincides with the geometric center of the trunk. The coordinate system of the *i*-th joint is denoted as *O_i_*–*X_i_Y_i_Z_i_*. The contact points *P*_1_ and *P*_2_ between the two manipulators and the space station’s inner wall may not be in the same plane, assuming *P*_1_ and *P*_2_ are fixed in a fixed coordinate system. Moreover, there may be obstacles on the inner wall. When the two manipulators of the robot detach from the inner walls of the space station, the robot enters the flight phase.

#### 2.2.1. Mathematical Model of the Robot in the Contact Phase

Firstly, the kinematics of the robot is analyzed. The trajectory equations for *O*_1_*O*_2_ and *O*_2_*O*_3_ can be formulated as

(1) $$\left(\begin{array}{ccc} S_{srx}^{p} & S_{sry}^{p} & S_{srz}^{p} \end{array}\right)=a_{sr}t_{r}^{3}+b_{sr}t_{r}^{2}+c_{sr}t_{r}+o_{sr}\;\;r=1,2$$

where *t*_1_ and *t*_2_ represent the times in the *r*-th phases of approaching the inner wall (*O*_1_*O*_2_ phase) and moving away from the inner wall (*O*_2_*O*_3_ phase), respectively. ***a****_s__r_*, ***b****_s__r_*, and ***c****_s__r_* represent the polynomial coefficients. ***o****_s_*_1_ represents the coordinate of the point *O*_1_, which is a known value. ***o****_s_*_2_ represents the coordinate of the point *O*_2_. While ***o****_s_*_2_ is on the trajectory *O*_1_*O*_2_, its position remains uncertain. It can be expressed as a function of position change Δ*y_os_*_2_, which is the distance between the ideal closest point and the actual closest point in the *Y*_0_ direction. The function can be written as

(2) $$o_{s2}=f\left(\Delta y_{os2}\right)$$

The trajectory *O*_2_*O*_3_ is variable and lies on the line between the optimized point *O*_2_ and the predetermined position point *O*_1_ for the subsequent cycle. By optimizing the polynomial coefficients and the position of ***o****_s_*_2_, the trajectory of the robot’s trunk can be determined. The velocity of the trunk can be obtained by deriving Equation (1).

Given that the robot needs to meet the expected movement, some values are known during the robot’s continuous movement, which results in ***a****_sr_*, ***b****_sr_*, and ***c****_sr_* not being independent. The known value ***U****_sr_* can be expressed as

(3) $$U_{sr}=\left\{P_{O1},v_{1},v_{2},v_{3}\right\}$$

where *P_O_*_1_ is the position of the trunk at point *O*_1_. *v*_1_, *v*_2_ and *v*_3_ are the velocities of the trunk at point *O*_1_, *O*_2_ and *O*_3_, respectively. By incorporating the aforementioned known values as boundary conditions into the position and velocity equations, the polynomial coefficients ***b****_sr_* and ***c****_sr_* can be expressed as functions of ***a****_s_*_2_ and Δ*y_os_*_2_.

The change in the trunk posture can be expressed as

(4) $$\left\{\begin{array}{l} \Theta_{srx}^{p}=a_{\Theta rx}t_{r}^{3}+b_{\Theta rx}t_{r}^{2}+c_{\Theta rx}t_{r}+\Theta_{xr}\\ \Theta_{sry}^{p}=a_{\Theta ry}t_{r}^{3}+b_{\Theta ry}t_{r}^{2}+c_{\Theta ry}t_{r}+\Theta_{yr}\\ \Theta_{srz}^{p}=a_{\Theta rz}t_{r}^{3}+b_{\Theta ry}t_{r}^{2}+c_{\Theta rz}t_{r}+\Theta_{zr} \end{array}\right.\;\;r=1,2$$

where *a*_Θ_, *b*_Θ_, and *c*_Θ_ represent the polynomial coefficients that can be determined through optimization in the *r*-th phases (*O*_1_*O*_2_ phase or *O*_2_*O*_3_ phase). (Θ*_x__r_*, Θ*_y__r_*, Θ*_z__r_*) is the initial posture of the trunk. The angular velocity of the trunk can be obtained by deriving Equation (4).

Similarly, due to the presence of boundary conditions, the polynomial coefficients *a*_Θ_, *b*_Θ_, and *c*_Θ_ in Equation (4) are not independent. The known value ***U***_Θ*r*_ can be expressed as

(5) $$U_{\Theta r}=\left\{\Theta_{1},\Theta_{2},{\dot{\Theta }}_{1},{\dot{\Theta }}_{2},{\dot{\Theta }}_{3}\right\}$$

where Θ_1_ and Θ_2_ are the postures of the trunk at points *O*_1_ and *O*_2_, respectively. ${\dot{\Theta }}_{1}$, ${\dot{\Theta }}_{2}$, and ${\dot{\Theta }}_{3}$ are the angular velocity of the trunk at points *O*_1_, *O*_2_, and *O*_3_, respectively. By introducing the known values as boundary conditions into the posture and angular velocity equations, the variables only consist of *a*_Θ2*x*_, *a*_Θ2*y*_, and *a*_Θ2*z*_.

Moreover, the kinematics modeling of the manipulators is established based on the analysis of the trunk parameters. The homogeneous transformation matrix of the trunk coordinate system *O_p_*–*X_p_Y_p_Z_p_* relative to the fixed coordinate system can be expressed as

(6) Tp0j=T10T21⋯Tii−1⋯Tpn−1

where Tii−1 is the homogeneous transformation matrix of the *i*-th coordinate system relative to (*i* − 1)-th coordinate system. The kinematics modeling of each manipulator involves six unknown joint angles *θ_i_* (*i* = 1, …, 6), which can be determined once the position and posture of the trunk are known. The joint angles, angular velocities, and angular accelerations can be formulated as

(7) $$\left[\begin{array}{ccc} \theta_{i}^{j} & {\dot{\theta }}_{i}^{j} & {\ddot{\theta }}_{i}^{j} \end{array}\right]=f_{1}\left(T_{p},t_{i},P_{i}\right)$$

where ***T****_p_* refers to the trunk parameters, including the position, velocity (angular velocity), and acceleration (angular acceleration) of the trunk. ***P****_i_* represents the position of the contact points between the manipulators and the inner wall.

The angular velocity and angular acceleration of the link can be expressed as

(8) ωiij=Ri−1iωi−1i−1j+θ˙ijQ^iαiij=Ri−1iαi−1i−1j+Ri−1iωi−1i−1j×θ˙ijQ^i+θ¨ijQ^i

where Ri−1i is the transformation matrix of the (*i* − 1)-th link relative to the *i*-th link, and ${\hat{Q}}_{i}$ is the direction vector.

The acceleration aiij of the origin of the *i*-th moving coordinate system for the *j*-th manipulator and the acceleration aicij of the center of mass of the *i*-th link can be expressed as

(9) aiij=Ri−1iαi−1i−1j×Pi−1ij+ωi−1i−1j×ωi−1i−1j×Pi−1ij+θ¨ijQ^iaicij=αiij×Picij+ωiij×ωiij×Picij+aiij

where Pi−1ij represents the direction vector of the origin of the *i*-th coordinate system relative to the origin of the (*i* − 1)-th coordinate system, and Picij represents the direction vector of the center of mass of the *i*-th link in the *i*-th coordinate system. ωiij represents the angular velocity of the link. Subsequently, the inertia force and inertia moment of each link of the manipulator can be expressed as

(10) FiIk=mijaicijMiIi=Iiijαiij+ωiij×Iiijωiij

where Iiij is an inertial tensor in the *i*-th coordinate system, which can be written as

(11) Iiij=∭m(yici2+zici2)dm−∭mxiciyicidm−∭mxicizicidm−∭mxiciyicidm∭m(xici2+zici2)dm−∭myicizicidm−∭mxicizicidm−∭myicizicidm∭m(xici2+yici2)dm

where xici, yici, and zici represent the coordinates of the center of mass of *i*-th link in the *i*-th coordinate system. Following this, the Newton–Euler method can be employed to calculate the mutual forces and torques between the links and the joint driving torques. For the *i*-th link, the equation can be written as

(12) Fiij−Ri+1iFi+1i+1j=FiIijMiij−Ri+1iMi+1i+1j−Picij×FiIij−Pii+1j×Ri+1iFiij=MiIij

where Fiij and Miij represent the constraint force and constraint torque of the (*i* − 1)-th link to the *i*-th link in the *i*-th coordinate system, respectively. The manipulators possess a total of 12 DOFs and require 12 drives for the two manipulators. When two manipulators come into contact with the inner wall simultaneously, the trunk has 6 DOFs, resulting in redundant actuation. The robot has 11 components, with a total of 66 equations and 72 unknowns. Optimization is necessary to find the optimal solution. To simplify the calculation, three joints for each manipulator are selected, and the driving torque variation is modeled as a polynomial.

(13) τiirj=aMirti3+bMirti2+cMir

where *a_Mir_*, *b_Mir_*, and *c_Mir_* are the polynomial coefficients in the *r*-th phase. By optimizing these coefficients, the driving torques of three selected joints for each manipulator can be obtained, and the driving torques of other joints can be obtained by solving Equation (12).

#### 2.2.2. Mathematical Model of the Robot in the Flight Phase

To ensure the robot’s trunk trajectory stays within the workspace when both manipulators make contact with the inner wall in the subsequent cycle, the angular velocity of the trunk is typically non-zero during the flight phase. This angular velocity depends on the posture of the trunk at point *O*_3_, the posture of the trunk at point *O*_1_ in the next cycle, and the duration of the flight phase. The duration *t_lk_* can be expressed as

(14) $$t_{lk}=s_{lk}^{p}/v_{O3k}$$

where *v_O_*_3*k*_ represents the velocity of the robot when it is at point *O*_3_ in the *k*-th cycle, and $s_{lk}^{p}$ represents the distance between point *O*_3*k*_ and point *O*_1(*k*+1)_. The angular velocity of the trunk can be written as

(15) $$\left\{\begin{array}{c} {\dot{\Theta }}_{lxk}^{p}=\Theta_{lxk}^{p}/t_{lk}\\ {\dot{\Theta }}_{lyk}^{p}=\Theta_{lyk}^{p}/t_{lk}\\ {\dot{\Theta }}_{lzk}^{p}=\Theta_{lzk}^{p}/t_{lk} \end{array}\right.$$

where $\Theta_{lxk}^{p}$, $\Theta_{lyk}^{p}$, and $\Theta_{lzk}^{p}$ are the rotation angles of the trunk during the flight phase. These can be obtained based on the posture vector $\vec{O_{3k}}$ of the trunk at point *O*_3*k*_ in the previous cycle and the posture vector $\vec{O_{1(k+1)}}$ of the trunk at point *O*_1(*k*+1)_ in the next cycle. The rotation matrix of vector $\vec{O_{3k}}$ to vector $\vec{O_{1(k+1)}}$ can be recorded as RO3kO1(k+1). The rotation angle of the trunk during the flight phase can be formulated as

(16) $$\left\{\begin{array}{l} \Theta_{lxk}^{p}=\arctan2\left(c_{32},c_{33}\right)\\ \Theta_{lyk}^{p}=\arctan2\left(-c_{31},\sqrt{c_{32}^{2}+c_{33}^{2}}\right)\\ \Theta_{lzk}^{p}=\arctan2\left(c_{21},c_{11}\right) \end{array}\right.$$

where *c_mn_* represents the element corresponding to the *m*-th row and *n*-th column of the matrix RO3kO1(k+1). Given the uncertainty of the position of the point *O*_2*i*_, the rotation angle of the trunk needs to be determined based on the optimization results.

To ensure the two manipulators of the robot maintain the expected posture at the moment of contact with the inner wall, the joint angles of the two manipulators are not constant during the flight phase. The expected posture can be written as

(17) $$\theta_{o1\left(i+1\right)}^{j}=f_{2}\left(T_{p},P_{i}\right)$$

where $\theta_{o1\left(i+1\right)}^{j}$ is the angle of the *j*-th joint at point *O*_1(*i*+1)_. The joint angular velocity can be expressed as

(18) $${\dot{\theta }}_{o1\left(i+1\right)}^{j}=\left.\left|\theta_{o3i}^{j}-\theta_{o1\left(i+1\right)}^{j}-\Delta \theta^{j}\right|/t_{li}\right|d_{p}\in D_{p}$$

where $\theta_{o3i}^{j}$ is the angle of the *j*-th joint at points *O*_3*i*_. $\Delta \theta^{j}$ is the adjustment coefficient. Moreover, the obstacle avoidance index *d_p_* of the robot should meet the constraint *D_p_*, indicating that the two manipulators must avoid colliding with each other during the movement process.

In summary, the establishment of the kinematics and dynamics models of the robot through the aforementioned process lays a foundation for subsequent parameter optimization.

### 2.3. Motion Parameters Optimization

#### 2.3.1. Constraints Analysis

(1) The continuous movement of a robot is governed by various constraints, including obstacle constraints, prohibited contact area constraints, and performance constraints.

Obstacle constraints: The two manipulators may not only collide with obstacles, but may also readily collide with each other, leading to joint–joint, joint–link, and link–link collisions. Collision detection is executed by simplifying the joints and links of the robot into capsules and obstacles into cuboids, followed by calculating the minimum distance between them. The self-collision detection is performed by calculating the minimum distance *d_qs_* between capsules, while the minimum distance *D_q_* between capsules and each plane of the cuboid is calculated to identify potential collisions with obstacles. The minimum distances *d_qs_* and *D_q_* should meet the following conditions:

(19) $$\left\{\begin{array}{c} d_{qs}>r_{q}+r_{s}+\Delta d\\ D_{q}>r_{q}+\Delta d \end{array}\right.$$

where *r_q_* and *r_s_* are the radii of the capsules, and Δ*d* is the safe distance.

(2) Prohibited contact area constraints: Within the space station, certain regions of the inner wall may be vulnerable and require protection from potential damage caused by contact with the robot. Therefore, it is necessary to avoid contact with such areas, which can be expressed as

(20) $$\left.P_{Ak}\notin H_{k}\right|H_{k}=(S_{Akx}\pm r_{A},S_{Akz}\pm r_{A})$$

where *S_Akx_* and *S_Aky_* represent the length and width of a rectangular prohibited contact area, respectively, and *r_A_* is the radius of the circular contact surface at the end of the manipulator.

(3) Performance constraints: The performance indices of the robot include motion feasibility, zero moment point (ZMP), total inertia moment, and energy consumption.

Motion feasibility: The motion feasibility constraint involves two aspects: firstly, the rotation angles of the joints of both manipulators and the trunk should be within the predefined ranges; secondly, the trajectories *O*_1_*O*_2_ and *O*_2_*O*_3_ of the trunk should lie within the workspace when two manipulators come into contact with the inner wall. These constraints can be expressed as

(21) $$\begin{array}{cc} \left\{\begin{array}{l} \theta_{i}^{j}\in \left[\theta_{i-\min}^{j},\theta_{i-\max}^{j}\right]\\ \Theta_{s}^{p}\in \left[\Theta_{s-\min}^{p},\Theta_{s-\max}^{p}\right] \end{array}\right. & and\;P_{p}\in C_{p} \end{array}$$

where $\left[\theta_{i-\min}^{j},\theta_{i-\max}^{j}\right]$ denotes the allowable range of joint angles and $\left[\Theta_{s-\min}^{p},\Theta_{s-\max}^{p}\right]$ is the allowable range of trunk posture. *P_t_* represents the set of discrete points of the position of the center of mass of the trunk, and *C_t_* denotes the workspace.

ZMP. To ensure the motion stability of the robot, ZMP is introduced as a performance index. When the gravity acceleration is not considered, ZMP can be expressed as

(22) XZMP=∑j=12∑i=16mijR0ijaicizjx0cij−∑j=12∑i=16mijR0ijaicixjz0cij∑j=12∑i=16mij(R0ijaicizjZZMP=0YZMP=∑j=12∑i=16mij(R0ijaicizjy0cij)−∑j=12∑i=16mij(R0ijaiciyjz0cij)∑j=12∑i=16mijR0ijaicizj

where $m_{i}^{j}$ represents the mass of the *i*-th link. x0cij,y0cij,z0cij and aicixj,aiciyj,aicizj represent the coordinates and the acceleration of the center of mass of each link in their respective coordinate systems. The stability of the robot can be indicated by the proximity between the ZMP and straight line *P*_1_*P*_2_. The distance can be expressed as

(23) $$d_{ZMP}=\left(\left|Ax_{ZMP}+By_{ZMP}+C\right|/\sqrt{A^{2}+B^{2}}\right)/P_{1}P_{2}$$

where *A*, *B,* and *C* are the coefficients of the linear equation.

Total inertia moment. The robot may exhibit large velocities and acceleration during continuous movement. Moreover, due to the frequent changes in the motion direction of the robot and its zero velocity at the point closest to the inner wall, the amplitude of velocity and acceleration can be substantial. This can result in significant changes in inertia torque. Excessive inertia torque and large amplitude can make the dynamic performance of the robot challenging to control. Therefore, changes in total inertia moment should stay within a reasonable range. The total inertia moment can be expressed as

(24) MG=∑j=12∑i=16P0ij×F0Iij+M0Iij+0Pt×F0It+M0It

where F0Iij and F0It represent the inertia forces of the *i*-th link of the *j*-th manipulator and the trunk, respectively. P0ij and P0t are the vectors of the center of mass of the *i*-th link of the *j*-th manipulator and the trunk in the fixed coordinate system, respectively. M0Iij and ^0^***M**_I__t_* are the inertia moments. The total inertia moment should be constrained from three aspects: mean, variance, and the total inertia moment at the moment of detachment from the inner wall. The mean value should also be within a reasonable range and not too large. It can be expressed as

(25) $$mean\left(M_{G}\right)\in \left[M_{G,\min}^{mean},M_{G,\max}^{mean}\right]$$

where *mean*(*M_G_*) represents the mean of the total inertia moment and $\left[M_{G,\min}^{mean},M_{G,\max}^{mean}\right]$ is the allowable range. The variance of the total inertia moment should be as small as possible to reduce control difficulty and load on the driving motors. The total inertia moment at the moment of detachment from the inner wall should also be minimized to ensure the robot’s stable movement during the flight phase.

Energy consumption. Energy consumption is another critical performance index for robots operating in a space station, where energy resources are limited. Therefore, it is essential to minimize energy consumption. The total energy consumption can be expressed as

(26) E=∫0T∑j=12∑i=16Pijtdt=∫0T∑j=12∑i=16τiijtθ˙ijtdt

where *P_ij_* denotes the instantaneous power of the *i*-th joint of the *j*-th leg, τiij is the joint torque, and ${\dot{\theta }}_{i}^{j}$ is the joint angular velocity.

#### 2.3.2. Optimize Variables and Objective Function

The optimization variables primarily consist of the trunk movement law, the contact points between manipulators and the inner wall, and the driving torques.

(1) Trunk movement law: Given the trajectory is not unique and the posture is not constant, the optimization variables include polynomial coefficient *a_s_*_2_ in Equation (1), polynomial coefficients *a_Θ_*_2*x*_, *a_Θ_*_2*y*_, and *a_Θ_*_2*z*_ in Equation (4), and Δ*y_os_*_2_ in Equation (2). Furthermore, the optimization variables also include the motion time *t*_1_ and *t*_2_ of the robot in the *O*_1_*O*_2_ and *O*_2_*O*_3_ phases.
(2) Contact points: The coordinates *P*_1_ = (*x_P_*_1_, *y_P_*_1_) and *P*_2_ = (*x_P_*_2_, *y_P_*_2_) of the two contact points are also optimized.
(3) Driving torques: The polynomial coefficients *a_Mp_*, *b_Mp_*, and *c_Mp_* in Equation (13), which represent the driving torques, are also considered optimization parameters.

The optimization objective function for the continuous movement of the robot can be expressed as

(27) $$\begin{array}{l} \underset{x\in Z_{0}}{G\left(x\right)}=\sum_{n=1}^{5}q_{n}G_{n}\left(x\right)\\ s.t.\;W \end{array}$$

where

$$G_{1}=\left(\left|mean\left(d_{ZMP}\right)\right|-mean\left|mean\left(d_{ZMP}\right)\right|\right)/\sqrt{\mathrm{var}\left|mean\left(d_{ZMP}\right)\right|};$$

$$G_{2}=\left(\left|\mathrm{var}\left(d_{ZMP}\right)\right|-mean\left|\mathrm{var}\left(d_{ZMP}\right)\right|\right)/\sqrt{\mathrm{var}\left|\mathrm{var}\left(d_{ZMP}\right)\right|};$$

$$G_{3}=\left(\mathrm{var}\left|M_{G}\right|-mean\left(\mathrm{var}\left|M_{G}\right|\right)\right)/\sqrt{\mathrm{var}\left(\mathrm{var}\left|M_{G}\right|\right)};$$

$$G_{4}=\left(End\left(\left|M_{G}\right|\right)-mean\left(\left|M_{G}\right|\right)\right)/\sqrt{\mathrm{var}\left(mean\left|M_{G}\right|\right)};$$

$$G_{5}=\left(E-mean\left(E\right)\right)/\sqrt{\mathrm{var}\left(E\right)}$$

where *G*_1_ and *G*_2_ symbolize the mean and variance of the discrete points corresponding to *d_ZMP_*. *G*_3_ and *G*_4_ represent the variance of the discrete points corresponding to the total inertia moment and the total inertia moment at the moment of detachment from the inner wall, respectively. *G*_5_ represents the energy consumption of the robot. *q_n_* is the weight coefficient. For the driving torques, the main optimization objective is energy consumption, represented by *G*_5_ in Equation (27).

#### 2.3.3. Optimization Method

**Figure 3:**
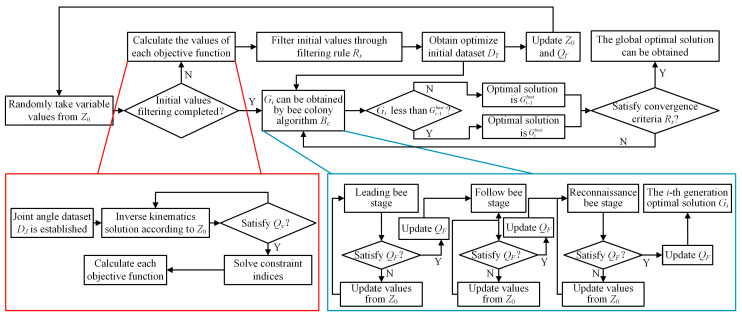
Optimization flowchart.

In the continuous movement process, a multitude of optimization parameters are involved. To enhance the optimization efficiency, an optimization algorithm based on an artificial bee colony algorithm is proposed, and the optimization flowchart is shown in Figure 3.

The optimization process begins by defining the initial range of optimization parameters *Z*_0_ = [*Z*_0-min_, *Z*_0-max_] and the initial constraint range *Q_F_* = [*Q_F_*_min_, *Q_F_*_max_] for the objective functions. Values from *Z*_0_ are randomly selected to ascertain if the initial dataset has been filtered. If it has not, the values of each objective function are calculated. During this calculation process, the precision and efficiency of the inverse kinematics of the manipulator are linked to the given initial values. The ranges of the two contact points are divided into equidistant grids. When the trunk of the robot is at point *O*_1_, the joint angles of the two manipulators, which correspond to the end of the manipulators at each grid point, are calculated. This process establishes the initial joint angle dataset, *D_J_*. To solve inverse kinematics, joint angles corresponding to the coordinates of the contact points closest to the target contact points can be selected from the dataset as the initial values. This significantly enhances the efficiency of the solving process. After solving the inverse kinematics, the constraint *Q_y_* is assessed to determine whether it is satisfied. If *Q_y_* is not met, the value is re-evaluated from *Z*_0,_ and the inverse kinematics is re-solved. If *Q_y_* is satisfied, each objective function value and its corresponding argument parameters are stored. Subsequently, high-quality data are selected from the initial values to form the optimized initial value dataset *D_Y_*, after which *Z_0_* and *Q_F_* are updated.

The filtered dataset *D_Y_* is then input into the artificial bee colony algorithm *B_c_* for further optimization. In the three stages of leading bee, following bee, and investigating bee, the solved objective function values are evaluated against the objective function range constraint *Q_F_*. If *Q_F_* is not met, the corresponding date set is discarded. If *Q_F_* is satisfied, it is updated, and the optimization process continues. At each generation of the artificial bee colony algorithm, the minimum multi-objective function value *G_i_* is compared with the current optimal solution $G_{i-1}^{best}$. If *G_i_* is superior to $G_{i-1}^{best}$, it is taken as the current global optimal solution. Otherwise, the current optimal solution remains $G_{i-1}^{best}$. After each generation of calculations, the convergence criteria *R_S_*, $G_{i+25}^{best}-G_{i}^{best}\leq 10^{-6}$, is checked. If *R_S_* is not met, the optimization process continues; otherwise, the current optimal solution is regarded as the global optimal solution. This optimization method can also be applied to optimize the driving torques.

## 3. Results

### 3.1. Calculation Results Analysis

To demonstrate the effectiveness of the proposed method, an example is presented. The structural parameters of two identical manipulators are shown in Table 1. Each link of the manipulator is assumed to have a mass of 0.05 kg/mm, while the trunk has a mass of 45 kg. The cross-section of the manipulator link is cylindrical with a radius of 300 mm, and that of the trunk link is rectangular with dimensions of 0.6 m × 0.8 m.

**Figure 4:**
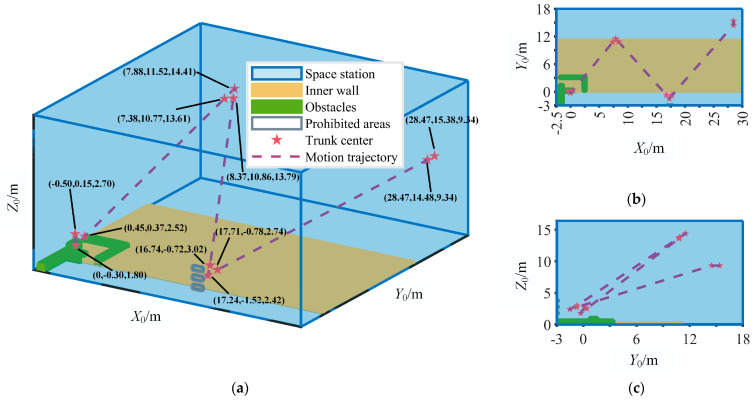
Motion trajectory of the robot’s trunk. (**a**) Axonometric map of motion trajectory. (**b**) Projection of motion trajectory on the *X*_0_*Y*_0_ plane. (**c**) Projection of motion trajectory on the *Y*_0_*Z*_0_ plane.

Suppose the space station model is a cuboid with dimensions of 32.5 m × 23.7 m × 18.6 m, and the fixed coordinate system of the robot during its first cycle as a reference. As depicted in Figure 4a, there are multiple obstacles within the region defined by points (−2 m, −3 m, 0 m), (2.80 m, −3 m, 0 m), (2.80 m, 3.47 m, 0 m), and (−2 m, 3.47 m, 0 m) on the lower wall plane, with a height range of (0.65 m to 1 m). A rectangular boss is present on the lower wall plane, with vertices at (−2.50 m, 0 m, 0 m), (−2.50 m, 11.30 m, 0 m), (30 m, 11.30 m, 0 m), and (30 m, 0 m, 0 m), respectively. This boss has a height of 0.3 m. On the front wall plane, there is a prohibited contact area where precision electronic components are located, situated within a rectangular region defined by points (16.73 m, −3 m, −1.55 m), (16.73 m, −3 m, 4.03 m), (17.98 m, −3 m, 4.04 m), and (17.98 m, −3 m, 1.55 m). The proposed algorithm possesses general applicability and is not dependent on the shape of the space station or the positioning of obstacles and prohibited contact areas. The coordinates for point *O*_1_ during the four cycles are (−0.50 m, 0.15 m, 2.70 m), (7.38 m, 10.77 m, 13.61 m), (16.74 m, −0.72 m, 3.02 m) and (28.47 m, 14.48 m, 9.34 m), respectively. The given parameters are shown in Table 2. Here, *v*_1*i*_, *v*_2*i*_, and *v*_3*i*_ are the velocities of the trunk at points *O*_1*k*_, *O*_2*k*_, and *O*_3*k*_, respectively, while $\Theta_{s1k}^{p}$ and $\Theta_{s2k}^{p}$ are the posture of the trunk at points *O*_1*k*_ and *O*_2*k*_, respectively. Moreover, *ω*_1*k*_ and *ω*_2*k*_ are the angular velocity of the trunk at points *O*_1*k*_ and *O*_2*k*_. For the given constraints, Δ*d* in Equation (19) is 0.15 m, while *r_q_* = *r_s_* = 0.30 m. *S_Akx_* and *S_Akz_* in Equation (20) are 1.25 m and 2.48 m, respectively, and *r_A_* is 200 mm. The rotation angle range for the four joints at *A_j_*, *B_j_*, *C_j_*, and *F_j_* is [−90°, 90°]. The rotation angle range for the two joints at *D*_1_ and *E*_2_ and the rotation angle range for the two joints at *D*_2_ and *E*_1_ are [−180°, 0°] and [0°, 180°], respectively. Moreover, the range of posture angles of the trunk around both the *X*_0_-axis and *Y*_0_-axis in a fixed coordinate system is [−25°, 25°], while the range around the *Z*_0_-axis is [−45°, 45°]. The range for the mean of the total inertia moment is [−55 Nm, 55 Nm]. For the OA-ABC, the number of honey sources, as well as the leading bees and following bees, is 100, with the maximum number of iterations being 100. The expansion coefficient for the honey source search range is 1. The threshold value for the leading bee to become an investigating bee is set at 55. The range of the two contact points are {[−200 mm, 500 mm], [−2000 mm, −500 mm]} and {[200 mm, 500 mm], [500 mm, 2000 mm]}, respectively. The above range is divided into a 71 × 151 grid, with each unit being 10 mm. The machine spec used is as follows: the CPU model is EPYC 7742, with 64 physical cores and 128 GB of RAM (Random Access Memory). Based on the method proposed in this paper, the optimization results of motion parameters are shown in Table 3. The optimization results of driving torques are shown in Table A1 in Appendix A.

The motion trajectory of the center of mass of the robot’s trunk in the space station is shown in Figure 4. Despite the complexity of the aforementioned environment, the robot can achieve continuous movement across various inner walls of the space station, with no intervening pauses between adjacent cycles. In the first cycle, the robot moves from the lower wall plane to the upper wall plane, followed by movement from the upper wall plane to the front wall plane during the second cycle, and from the front wall plane to the rear wall plane in the third cycle. During the fourth cycle, the robot stops when it reaches its closest point to the inner wall. The coordinates of the contact points between the manipulators and the inner wall during the four cycles are shown in Table 3. The robot will encounter obstacles during the first cycle and prohibited contact areas during the third cycle.

**Figure 5:**
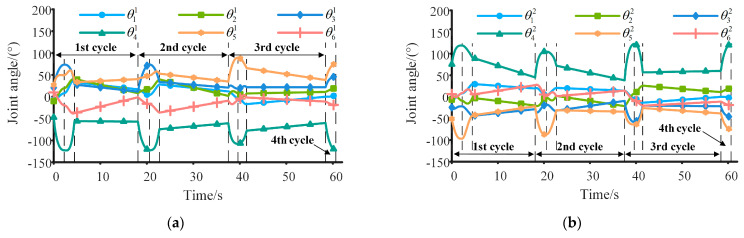

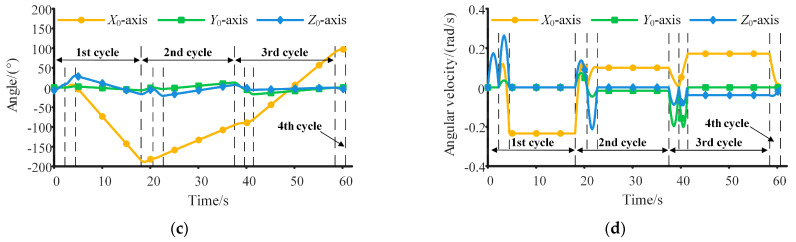
Motion law of the robot over four cycles. (**a**) The change in joint angles of manipulator 1. (**b**) The change in joint angles of manipulator 2. (**c**) The change in posture angle of the trunk. (**d**) The change in angular velocity of the trunk.

The variations in the joint angles of the two manipulators are shown in Figure 5a,b. Throughout the contact phases, the maximum angle range for the four joints at *A_i_*, *B_i_*, *C_i_*, and *F_i_* is [−37.74°, 73.83°], the maximum angle range for the two joints at *D*_1_ and *E*_2_ is [−122.71°, −47.01°], and the maximum angle range for the two joints at *D*_2_ and *E*_1_ is [26.37°, 122.58°]. The rotation angles remain within the permissible range. During the flight phase, as shown in Figure 5a,b, the joint angles of the manipulators undergo significant changes, with manipulator 1 and manipulator 2 reaching maximum joint rotation angles of 31.10° and 39.48°, respectively. The movement of the manipulators’ joints during this flight phase enables the robot to achieve the desired posture at the onset of the next cycle, ensuring movement continuity and superior performance. In terms of obstacle avoidance, during the contact phase, the minimum distance between the simplified capsules of two manipulators is 1.64 m, while the minimum distance between the capsules and the obstacles is 0.20 m. There are no collisions between the manipulators and obstacles. Similarly, during the flight phase, the minimum distance between the simplified capsules of the two manipulators is 1.48 m, which adequately satisfies the requirements for obstacle avoidance.

The variations in the angle and angular velocity of the trunk are presented in Figure 5c,d, respectively. As can be seen from Figure 5c, the posture of the trunk is not constant and exhibits a polynomial variation law during the contact phase, which is obtained by optimization. Across the four cycles, the maximum rotation angles of the trunk around the *X*_0_, *Y*_0,_ and *Z*_0_ axes are 9.58°, 7.00°, and 22.26°, respectively. If the robot’s trunk posture remains unchanged, it results in a significant increase in the calculated mean and variance of *d_ZMP_*, the variance of the total inertia moment and the total inertia moment at the moment of detachment from the inner wall, and the energy consumption of the robot, thereby leading to a decrease in overall performance.

**Figure 6:**
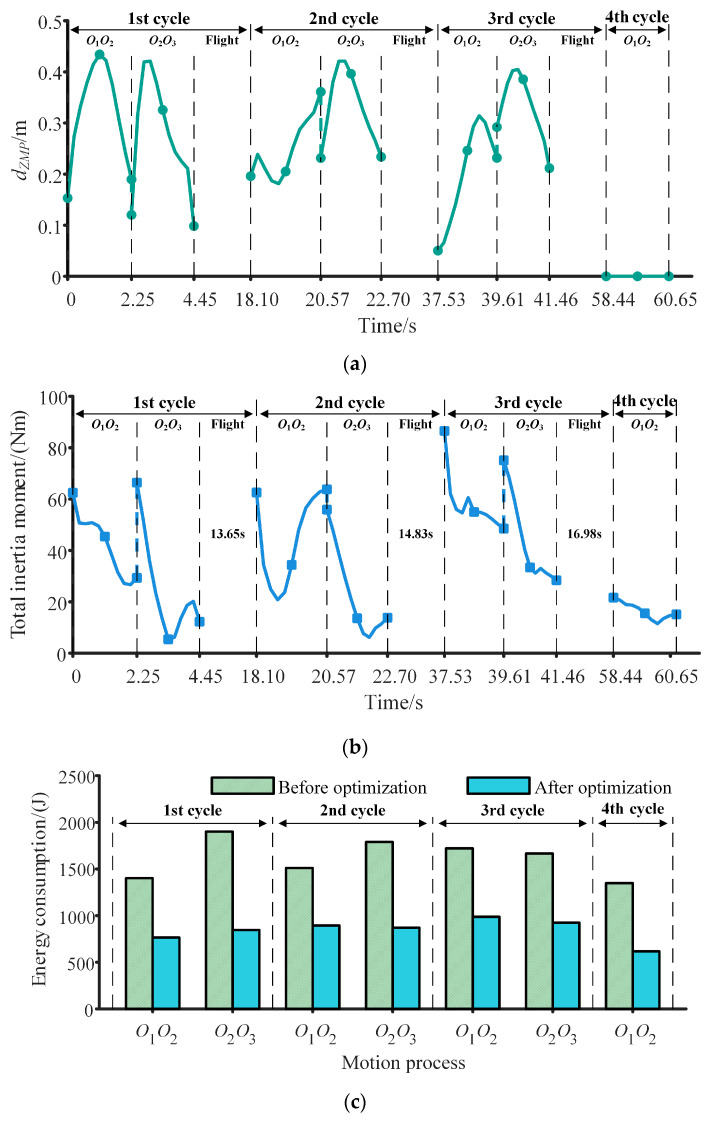
Changes in performance indices of robots in four cycles. (**a**) Changes in *d_ZMP_*. (**b**) Change in total inertia moment. (**c**) Changes in energy consumption.

The change in *d_ZMP_* is shown in Figure 6a. It can be seen from Figure 6a that *d_ZMP_* fluctuates within a minimal range, reaching maximum values of 0.43, 0.42, 0.40, and 0 during the four cycles, respectively. Notably, the sudden variation in *d_ZMP_* during the transition from the *O*_1_*O*_2_ phase to the *O*_2_*O*_3_ phase can be attributed to the change in acceleration direction, but this does not compromise the robot’s dynamic stability. By controlling the trajectory and posture of the trunk, *d_ZMP_* remains at zero during the fourth cycle. The change in total inertia moment is shown in Figure 6b, which exhibits a smooth variation. The robot possesses a relatively large total inertia moment at the beginning of the *O*_1_*O*_2_ phase to the *O*_2_*O*_3_ phase due to the high acceleration when the direction of movement changes. The mean values of the total inertia moment for the four cycles are 33.22 Nm, 33.97 Nm, 50.70 Nm, and 16.47 Nm, respectively, while the variances are 331.45 Nm, 407.66 Nm, 238.43 Nm, and 10.78 Nm, all within small ranges. At the moment of detachment from the inner wall, the total inertia moments are 12.29 Nm,13.84 Nm, and 28.40 Nm, which are close to zero. These results demonstrate that the robot possesses good dynamic stability. The changes in energy consumption are shown in Figure 6c. Prior to optimization, the robot’s energy consumption during the four cycles, using the initial optimization values as the known values, is 3303.27 J, 3299.09 J, 3388.37 J, and 1348.79 J, respectively. Following optimization, the energy consumption of the robot during the four cycles is reduced by 51.24%, 46.48%, 43.57%, and 54.24%, respectively. These findings satisfy the requirement for low energy consumption by robots within the space station.

**Figure 7:**
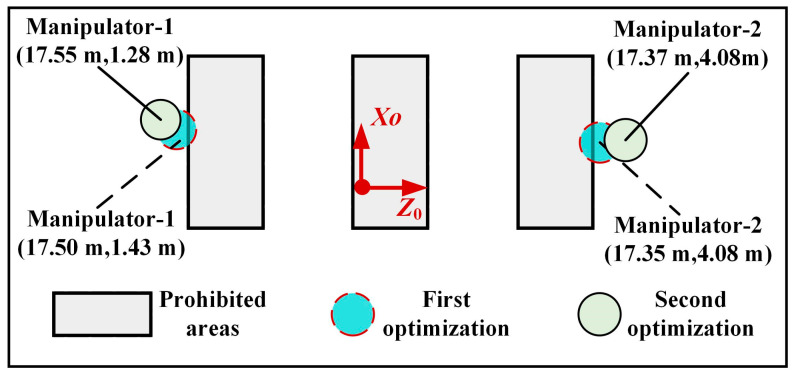
Diagram of prohibited areas and contact points between robot and inner walls.

Figure 7 shows the prohibited contact areas and contact points between robots and inner walls in the fixed coordinate system during the third cycle. The length and width sides of these areas are parallel to the *X*_0_-axis and *Z*_0_-axis, respectively. The positions of the centers of the three rectangles in the moving coordinate system for the third cycle are (17.35 m, 1.79 m), (17.35 m, 2.79 m), and (17.35 m, 3.78 m). Without considering the prohibited contact areas, the optimized positions of the two contact points are (17.50 m, 1.43 m) and (17.35 m, 4.08 m), respectively. However, these contact points fall within the prohibited contact areas. Thus, the contact position is optimized twice, resulting in new contact points at (17.55 m,1.28 m) and (17.37 m, 4.35 m), respectively. Analysis shows that the modified contact point positions have not significantly impacted the overall performance of the robot.

The results from the above analysis demonstrate that the method proposed in this paper enables the robot to achieve continuous movement among the various complex inner walls of the space station while maintaining good comprehensive performance. This provides a theoretical basis for the control of robots within a space station.

### 3.2. Simulation Results Analysis

To further substantiate the feasibility of the method proposed in this paper, a simulation is conducted. Software Webots is employed to simulate the examples discussed in Section 3.1. Webots is an open-source and multi-platform desktop application used to simulate various types of robots, including industrial manipulators and legged robots. We employ Webots purely for simulation purposes, making no modifications to the software itself, in compliance with the software license. Webots is capable of providing realistic dynamic simulation effects and can replicate complex environments, making it an ideal match for our research. During the simulation process, the structural parameters of the robot (including the size and weight) and environmental settings are aligned with the theoretical calculations. The friction between joints is disregarded. In addition to the known values shown in Section 2.2, the positions of the contact points and the driving torques are also provided as known values.

**Figure 8:**
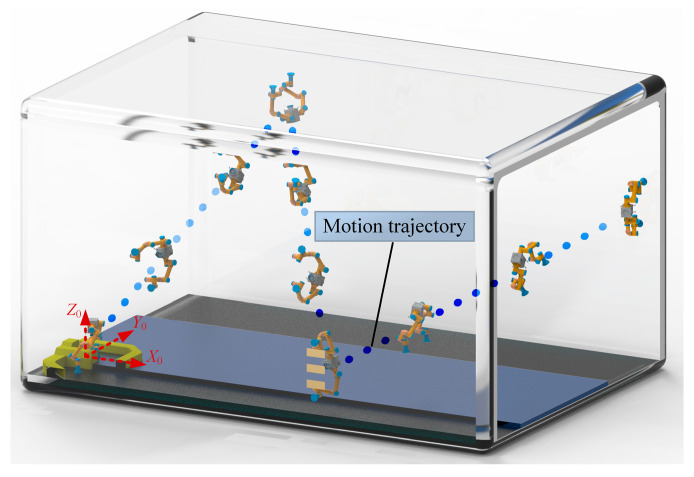
Simulation diagram of continuous motion of a dual-arm robot in a space station.

**Figure 9:**
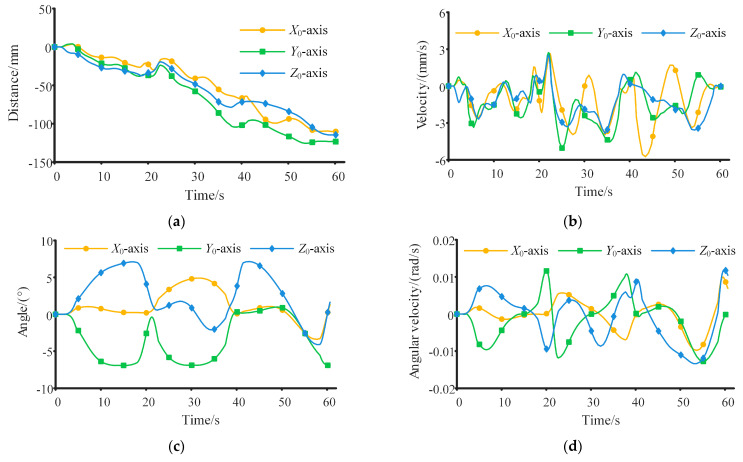
Difference between simulation results and theoretical calculation results. (**a**) The difference in motion trajectory. (**b**) The difference in trunk velocity. (**c**) The difference in trunk posture. (**d**) The difference in trunk angular velocity.

**Figure 10:**
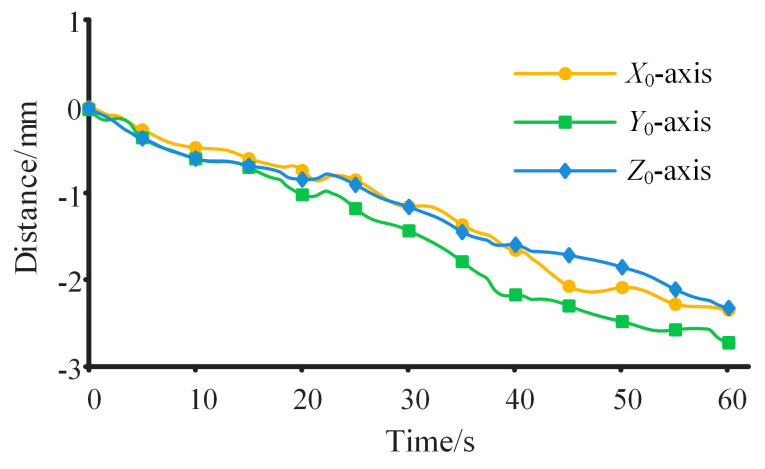
Difference in the motion trajectory of the trunk with and without considering joint friction.

The motion process of the robot within the space station is shown in Figure 8. It can be intuitively seen from Figure 8 that the motion trajectory and the change in the posture of the robot. The discrepancy between the theoretical calculation results and the simulation results for the motion law of the robot is shown in Figure 9. Figure 9a shows the difference in the motion trajectory of the robot. The cumulative errors result in an increasing trajectory difference. The difference in the motion trajectory along the three axes is 110.23 mm, 123.41 mm, and 110.51 mm at the end of the continuous movement, respectively. Compared with the total movement distance (60.32 m in four cycles), the error remains within a marginal range. Figure 9b shows the variation in the trunk velocities, with maximum and minimum values of 5.72 × 10^−3^ m/s and 0 m/s, respectively. Moreover, the ratio of the maximum difference to the maximum motion velocity of the robot is merely 0.01. Figure 9c,d show the difference between the theoretical calculation results and simulation results regarding the trunk angle and angular velocity, where the difference in both angle and angular velocity fluctuates within small ranges. The variation range for the angle difference is [−6.91°, 7.10°], and for angular velocity difference, it is [−0.013 rad/s, 0.011 rad/s]. These ranges also remain within a reasonable range when compared with the actual rotation angles and angular velocities of the robot’s trunk. The disparity between the theoretical calculation results and the simulation results can primarily be attributed to the following key factor. The inability to completely immobilize the contact points, and the micro-slip of the end of the manipulator relative to the inner wall, leads to changes in the motion law of the robot. Hence, during the prototype production stage, high-friction materials or adsorption devices can be employed at the end of the manipulators to prevent contact point slips. The aforementioned simulation was conducted without considering joint friction. However, joint friction could potentially influence omnidirectional continuous movement [44]. Assuming a friction coefficient of 0.15, the difference in the motion trajectory of the trunk, both with and without considering joint friction, is shown in Figure 10. It can be seen from Figure 10 that the trunk’s trajectory, after factoring in friction, deviates from the previous simulation trajectory. This deviation occurs because the presence of friction can alter the force distribution at the manipulator joints, thereby affecting the trunk’s trajectory. However, this deviation falls within a small range, with a maximum error of 2.7 mm. A larger friction coefficient may lead to a greater deviation in movement and increased energy consumption.

The aforementioned analysis indicates that the simulation results of the robot align closely with the theoretical calculation results, supporting the feasibility of the method proposed in this paper.

## 4. Discussion

The strengths of the algorithm proposed in this paper are primarily evident in the following areas.

(1) Traditional artificial bee colony algorithms randomly select honey sources within the range of independent variables. For our algorithm, we create an initial dataset prior to optimization. This means that the randomly obtained honey sources are screened based on the values of each objective function, leading to enhanced computational efficiency;
(2) The range of contact points between the manipulators and the inner wall is meshed, and a joint angle dataset is established. Using the joint angles corresponding to the mesh points closest to the actual contact points as the initial values effectively addresses the problem of low efficiency in solving the inverse kinematics of the manipulators;
(3) A single objective function range constraint is introduced in the three stages of leading bee, following bee, and investigating bee. This strategy allows for the elimination of some solutions that evidently do not meet the requirements even before calculating and comparing the normalized multi-objective function values;
(4) Throughout the optimization process, the range of independent variables in the initial dataset and the constraint range of each objective function are dynamically updated to improve the convergence speed of the algorithm.

**Figure 11:**
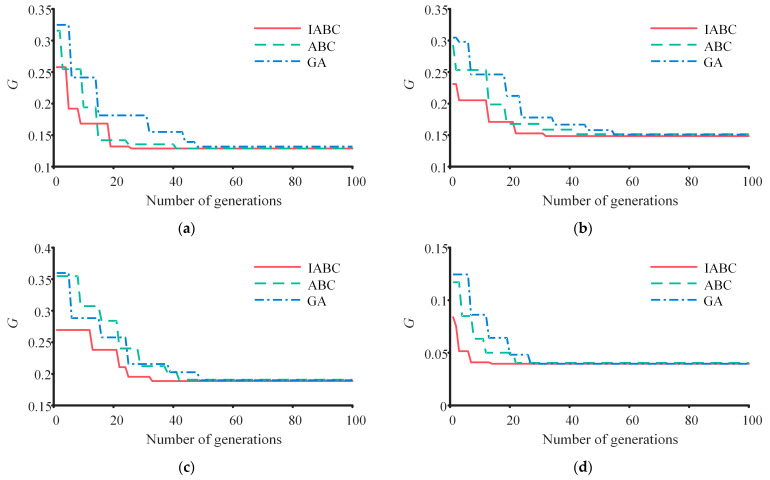
Convergence speed of optimization algorithms. (**a**) Cycle 1. (**b**) Cycle 2. (**c**) Cycle 3. (**d**) Cycle 4.

To further underscore the superiority of OA-ABC introduced in this paper, we compare it with the traditional artificial bee colony algorithm (ABC) and the genetic algorithm (GA) [45,46,47]. For the GA, we set the number of individuals (NIND) at 100, the maximum number of generations (MAXGEN) at 100, and the generation gap (GGAP) at 0.9. The convergence speeds of the algorithms are shown in Figure 11. The convergence algebra (cg), calculation time, and final objective function values of different algorithms are shown in Table 4. Both Figure 11 and Table 4 clearly demonstrate that the method proposed in this paper has the fastest convergence speed and is capable of delivering solutions that meet the requirements in the shortest possible time.

The comparison of the omnidirectional mobile robot proposed in this paper with existing space mobile robots is shown in Table 5. The humanoid robot, Rollin’ Justin, is a wheel-type mobile space robot capable of utilizing its two manipulators for collaborative tasks [14]. Chen et al. [15] developed a multi-toed quadruped robot, which is a typical bionic-legged robot. This type of robot exhibits good adaptability to complex terrains but has limited operational capabilities. Zhang et al. [18] developed a full-size and free-flying humanoid robot that can nearly replicate an astronaut’s movements, indicating promising application prospects. However, there is no mention of its capacity for omnidirectional continuous movement in the published research results. The space robot Astrobee, developed by Stanford University, features a straightforward structure, facilitating easy installation and placement. Nevertheless, its omnidirectional mobility requires further enhancement [19]. The robot proposed in this paper employs its two manipulators as both a mobile device and an operating device. The robot is capable of swiftly moving between different inner walls of the space station and exhibits good obstacle-crossing capabilities.

## 5. Conclusions

In order to facilitate efficient robot movement within a space station, this paper proposed an omnidirectional continuous movement method for a dual-arm robot. This method emulates the way astronauts use the reaction forces from their hands or feet and the inner walls to move around. (1) After determining the configuration of the robot’s two manipulators, mathematical models for both the contact and flight phases were established. These models clarified the relationship between motion parameters and dynamic performance, underscoring the significance of the robot’s motion mode in achieving continuous movement. (2) Several constraints were proposed, including obstacle constraints, prohibited contact area constraints, and performance constraints. After determining the optimization parameters, an optimization algorithm based on the artificial bee colony algorithm (OA-ABC) was proposed to enhance computational efficiency through the stepwise screening of variables and objective function values. (3) An example was provided of a robot achieving omnidirectional continuous movement within a space station model measuring 32.5 m × 23.7 m × 18.6 m. The robot achieves continuous movement between different inner walls over more than three cycles, covering a total distance of 60.32 m. During the flight phase, the maximum rotation angle and angular velocity of the robot’s trunk were 189° and 0.23 rad/s, respectively. In long-distance movement, the error between the theoretically calculated results and the simulation results fell within a reasonable range, and the robot demonstrated good comprehensive performance. These findings validate the feasibility of the method proposed in this paper. The method proposed in this paper provides a theoretical reference for controlling the motion of robots in a space station.

## Figures and Tables

**Table 1:** Structural parameters of the dual-arm robot.

*l*_11_/m	*l*_21_/m	*l*_31_/m	*l*_41_/m	*l*_51_/m	*l*_61_/m	*L*/m
0.20	0.40	1.20	0.40	1.20	0.40	0.52

**Table 2:** Known parameters in the continuous motion process of robot.

	*v*_1*k*_/(m/s)	*v*_2*k*_/(m/s)	*v*_3*k*_/(m/s)	Θ_1*k*_/°	Θ_2*k*_/°	ω_1*k*_/(rad/s)	ω_2*k*_/(rad/s)	ω_3*k*_/(rad/s)
Cycle 1	(0.5, −0.5, −1)	(0, 0, 0)	(0.5, 0.8, 0.8)	(0, 0, −7)	(0, 0, 8)	(0, 0,0)	(0, 0, 0)	(−0.23, 0, 0)
Cycle 2	(0.5, 0.8, 0.8)	(0, 0, 0)	(0.6, −0.8, −0.7)	(−175, −7, −17)	(−180, 0, −4)	(−0.23, 0, 0)	(0, 0, 0)	(0.1, 0, −0.013)
Cycle 3	(0.6, −0.8, −0.7)	(0, 0, 0)	(0.6, 0.4, 0.9)	(−95, −13, 7)	(−90, −3, 0)	(0.1, 0, −0.013)	(0, 0, 0)	(017, 0, −0.4)
Cycle 4	(0.6, 0.4, 0.9)	(0, 0, 0)	-	(90, 0, 0)	(97, −4, 0)	(017, 0, −0.4)	(0, 0, 0)	-

**Table 3:** Optimization results of motion parameters.

	*x*_*P*1_/mm	*y*_*P*1_/mm	*x*_*P*2_/mm	*y*_*P*2_/mm	Δ*y*_*os*2_/mm	*t*_1_/s	*t*_2_/s	*a*_*s*1_	*a*_*Θ*1*x*_	*a*_*Θ*1*y*_	*a*_*Θ*1*z*_
Cycle 1	122.20	−1150.23	285.70	1600.25	−300.03	2.25	2.20	0.050	−0.061	−0.010	−0.073
Cycle 2	110.27	−1150.41	299.40	1647.59	−306.70	2.47	2.13	0.044	−0.012	−0.014	0.065
Cycle 3	130.07	−1835.15	306.47	1238.42	95.84	2.08	1.85	0.034	−0.043	−0.043	0.079
Cycle 4	0	−1400	0	1400	0	2.21	-	-	-	-	-

**Table 4:** Comparison results of different optimization algorithms.

	OA-ABC			ABC			GA		
	cg	*t_s_*/h	*G*	cg	*t_s_*/h	*G*	cg	*t_s_*/h	*G*
Cycle 1	26	1.8	0.129	41	3.1	0.129	48	3.4	0.132
Cycle 2	32	2.3	0.148	55	3.5	0.151	42	3.3	0.151
Cycle 3	33	2.4	0.188	42	3.4	0.191	49	3.6	0.190
Cycle 4	14	1.1	0.040	22	1.7	0.041	27	2.0	0.040

**Table 5:** Comparison results of different space mobile robots.

	Omnidirectional Mobility	Collaborative Ability	Environmental Adaptability
Rollin’Justin [14]	×	√	↘
Multi-toed quadruped robot [15]	×	×	→
Taikobot [18]	Not mentioned	√	↗
Astrobee [19]	×	×	→
Proposed robot	↗	√	↗

↗: Advantage; →: Moderate; ↘: Inferiority; √: Existence; and ×: None.

## Data Availability

The original data contributions presented in the study are included in the article; further inquiries can be directed to the corresponding authors.

## Nomenclature

$S^{P}$	Position of center of mass of trunk
$\Theta^{P}$	Trunk posture
*a/b/c*	Polynomial coefficients
*θ/*$\dot{\theta }$/$\ddot{\theta }$	Joint angle/joint angular velocity/joint angular acceleration
ωiij/αiij	Link angular velocity/link angular acceleration
aiij/aicij	Joint acceleration/acceleration of the center of mass of the link
FiIk/MiIk/*M_G_*	Inertial force/inertia moment/total inertia moment
Iiij	Inertia tensor
*X_ZMP_*/*Y_ZMP_*	Coordinates of ZMP
***P***/***r***	Position vectors
Fiij/Miij/τiij	Force between links/torque between links/drive torque
*E*	Energy consumption
*A_j_* (*x_Aj_/y_Aj_*)	Landing point coordinates

## Appendix A

The driving torque contains a large number of optimization variables. For the example shown in Section 3.1, the optimization results of the parameters corresponding to the driving torque are shown in Table A1.

**Table A1:** Optimization results of driving torques.

Cycle 1	***a_M_*_11_**	***b_M_*_11_**	***c_M_*_11_**	***a_M_*_21_**	***b_M_*_21_**	***c_M_*_21_**	***a_M_*_31_**	***b_M_*_31_**	***c_M_*_31_**
	**−3.82**	5.24	74.98	10.67	−3.98	−85.38	−9.48	19.75	−140.76
	***a_M_*_41_**	***b_M_*_41_**	***c_M_*_41_**	***a_M_*_51_**	***b_M_*_51_**	***c_M_*_51_**	***a_M_*_61_**	***b_M_*_61_**	***c_M_*_61_**
	−11.91	11.47	−65.49	−1.45	−10.01	96.36	6.30	−14.17	−123.40
	***a_M_*_12_**	***b_M_*_12_**	***c_M_*_12_**	***a_M_*_22_**	***b_M_*_22_**	***c_M_*_22_**	***a_M_*_32_**	***b_M_*_32_**	***c_M_*_32_**
	−1.86	3.65	125.61	−7.77	10.19	73.10	10.87	−7.08	−95.5
	***a_M_*_42_**	***b_M_*_42_**	***c_M_*_42_**	***a_M_*_52_**	***b_M_*_52_**	***c_M_*_52_**	***a_M_*_62_**	***b_M_*_62_**	***c_M_*_62_**
	−14.03	−13.91	67.13	5.50	−0.22	59.92	10.09	−8.06	128.91
Cycle 2	***a_M_*_11_**	***b_M_*_11_**	***c_M_*_11_**	***a_M_*_21_**	***b_M_*_21_**	***c_M_*_21_**	***a_M_*_31_**	***b_M_*_31_**	***c_M_*_31_**
	10.87	−11.08	−54.42	−13.01	−2.45	−62.20	−3.91	−9.13	44.93
	***a_M_*_41_**	***b_M_*_41_**	***c_M_*_41_**	***a_M_*_51_**	***b_M_*_51_**	***c_M_*_51_**	***a_M_*_61_**	***b_M_*_61_**	***c_M_*_61_**
	−5.41	7.72	−131.23	2.84	5.99	−52.87	−18.45	2.24	15.01
	***a_M_*_12_**	***b_M_*_12_**	***c_M_*_12_**	***a_M_*_22_**	***b_M_*_22_**	***c_M_*_22_**	***a_M_*_32_**	***b_M_*_32_**	***c_M_*_32_**
	8.70	−2.09	97.83	−8.58	11.75	−64.39	−7.12	0.49	−66.28
	***a_M_*_42_**	***b_M_*_42_**	***c_M_*_42_**	***a_M_*_52_**	***b_M_*_52_**	***c_M_*_52_**	***a_M_*_62_**	***b_M_*_62_**	***c_M_*_62_**
	7.70	−10.62	−121.93	5.43	−7.16	−52.20	3.31	−2.23	117.40
Cycle 3	***a_M_*_11_**	***b_M_*_11_**	***c_M_*_11_**	***a_M_*_21_**	***b_M_*_21_**	***c_M_*_21_**	***a_M_*_31_**	***b_M_*_31_**	***c_M_*_31_**
	−1.82	−7.53	−57.41	5.30	16.26	−51.13	13.50	−3.98	−54.73
	***a_M_*_41_**	***b_M_*_41_**	***c_M_*_41_**	***a_M_*_51_**	***b_M_*_51_**	***c_M_*_51_**	***a_M_*_61_**	***b_M_*_61_**	***c_M_*_61_**
	−5.45	12.21	72.25	−2.24	15.11	−31.63	6.59	−6.35	−57.98
	***a_M_*_12_**	***b_M_*_12_**	***c_M_*_12_**	***a_M_*_22_**	***b_M_*_22_**	***c_M_*_22_**	***a_M_*_32_**	***b_M_*_32_**	***c_M_*_32_**
	−2.39	−4.05	−85.78	−10.98	1.49	45.54	12.35	3.20	57.83
	***a_M_*_42_**	***b_M_*_42_**	***c_M_*_42_**	***a_M_*_52_**	***b_M_*_52_**	***c_M_*_52_**	***a_M_*_62_**	***b_M_*_62_**	***c_M_*_62_**
	/13.34	−6.46	75.49	3.61	−14.28	14.99	7.91	12.95	89.65
Cycle 4	***a_M_*_12_**	***b_M_*_12_**	***c_M_*_12_**	***a_M_*_22_**	***b*_M22_**	***c_M_*_22_**	***a_M_*_32_**	***b_M_*_32_**	***c_M_*_32_**
	−7.91	5.63	−82.71	−5.78	−4.02	42.30	5.58	−0.76	33.95
	***a_M_*_42_**	***b_M_*_42_**	***c_M_*_42_**	***a_M_*_52_**	***b_M_*_52_**	***c_M_*_52_**	***a_M_*_62_**	***b_M_*_62_**	***c_M_*_62_**
	6.72	−1.53	75.49	0.89	1.64	41.82	−5.06	−1.56	−61.68
